# Biomechanics and indications for application of the method of BDSF. Answer to manuscript draft number AOTS-D- 17-00378, Letter to the Editor concerning ‘‘Femoral neck fracture osteosynthesis by the biplane double-supported screw fixation method (BDSF) reduces the risk of fixation failure: clinical outcomes in 207 patients’’ by Filipov O, Sommer C, et al. (2017) Arch Orthop Trauma Surg. Apr 8. [Epub ahead of print]

**DOI:** 10.1007/s00402-017-2716-9

**Published:** 2017-06-30

**Authors:** Orlin Filipov, Karl Stoffel, Boyko Gueorguiev, Christoph Sommer

**Affiliations:** 1 Vitosha Hospital, Simeonovsko Shose Str.108-B, 1700 Sofia, Bulgaria; 2 University Basel, Cantonal Hospital Baselland, Basel, Switzerland; 3 AO Research Institute Davos, Davos, Switzerland; 4 Cantonal Hospital Graubuenden, Chur, Switzerland

Dear Prof. Blauth:

We thank you very much for the reader’s Letter to the Editor and for all questions regarding the BDSF clinical application raised. Below we place our point-by-point answers.

## Biomechanical testing and biomechanics of the method of BDSF

Reader’s question 1. The reader suggests that “a comprehensive biomechanical study is necessary to demonstrate that the novel BDSF is better than the traditional reverse triangle screw fixation”.

**Fig. 1:**
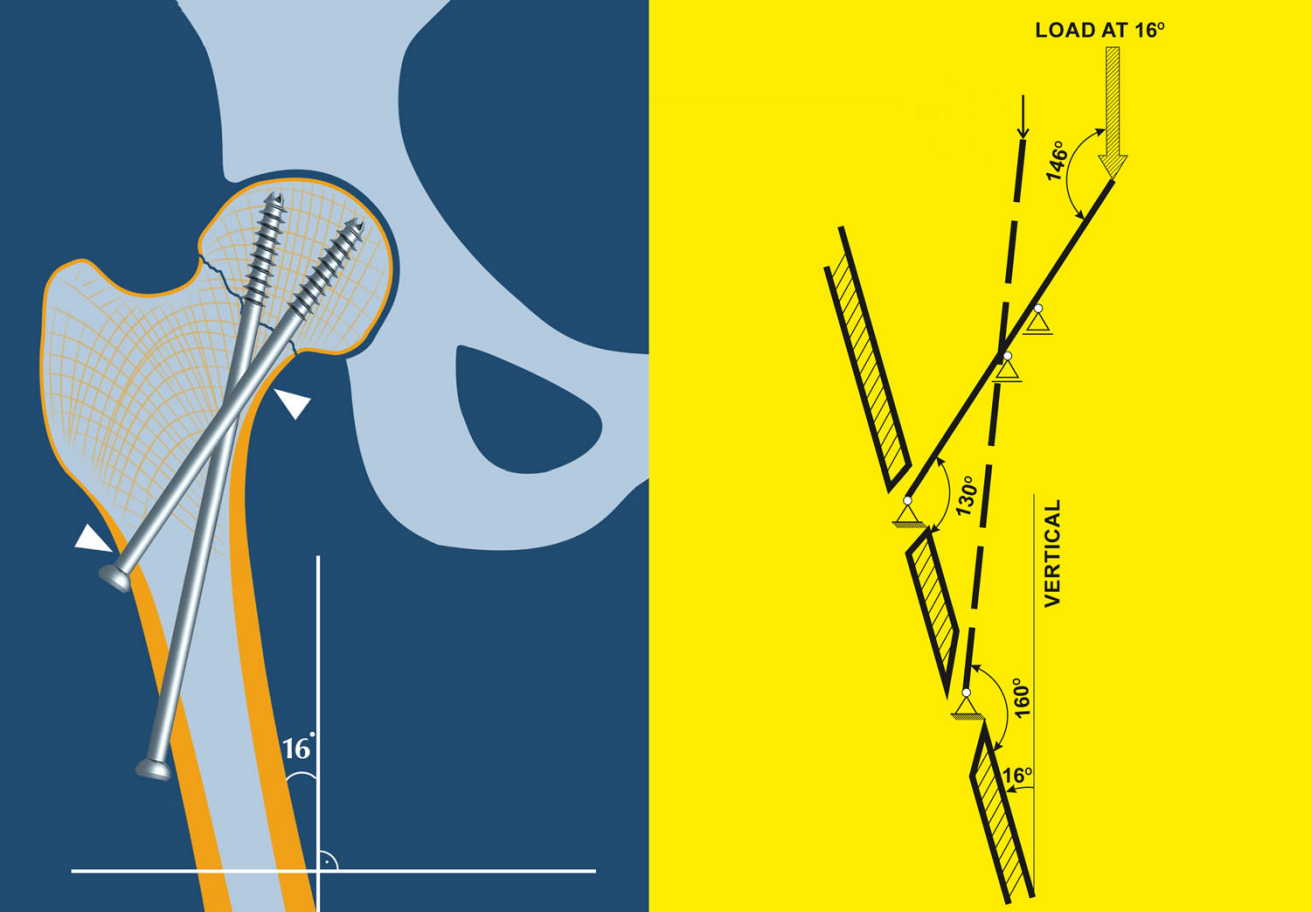
Biomechanical testing [1]. The applied load is *vertical* and the femoral shaft is inclined at 16° varus inclination to resemble the physiological resultant force inclination of 16° to the *vertical* in a standing position, according to Bergmann et al. [2]. The femoral neck and the middle BDSF screw which is parallel to the neck axis are in a more *vertical* orientation. The weight-bearing capacity of the middle BDSF screw is optimal and the shearing forces are smaller than would be in a more *vertical* varus inclination. Schematic representation of the middle and distal BDSF screws; the proximal BDSF screw is not shown

**Fig. 2:**
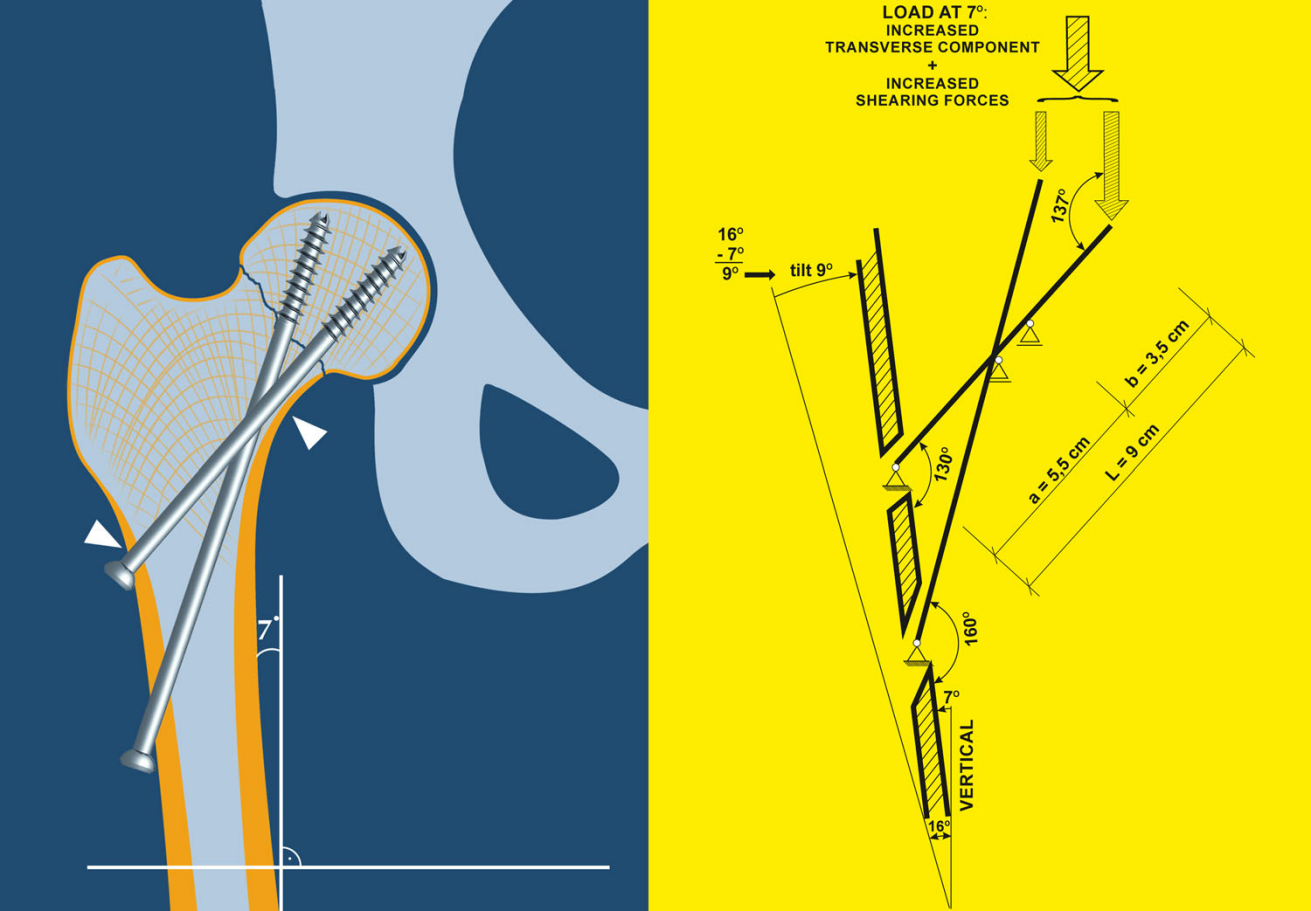
Biomechanical testing [1]. The applied load is *vertical* and the femoral shaft is inclined at 7° varus inclination to resemble the physiological resultant force inclination of 7° to the *vertical* when standing on one leg, according to Bergmann et al. [2]. The femoral neck and the middle BDSF screw which is parallel to the neck axis are in a more *horizontal* position. The *middle* BDSF screw weight-bearing capacity is significantly decreased and the shearing forces are increased in this more *vertical* position (7°) of the femur. The obtuse *distal* BDSF screw now is in optimal orientation for axial weight bearing. Its bearing capacity is added to the *middle* BDSF screw and helps maintain constant stability. Schematic representation of the middle and distal BDSF screws; the proximal BDSF screw is not shown

**Fig. 3:**
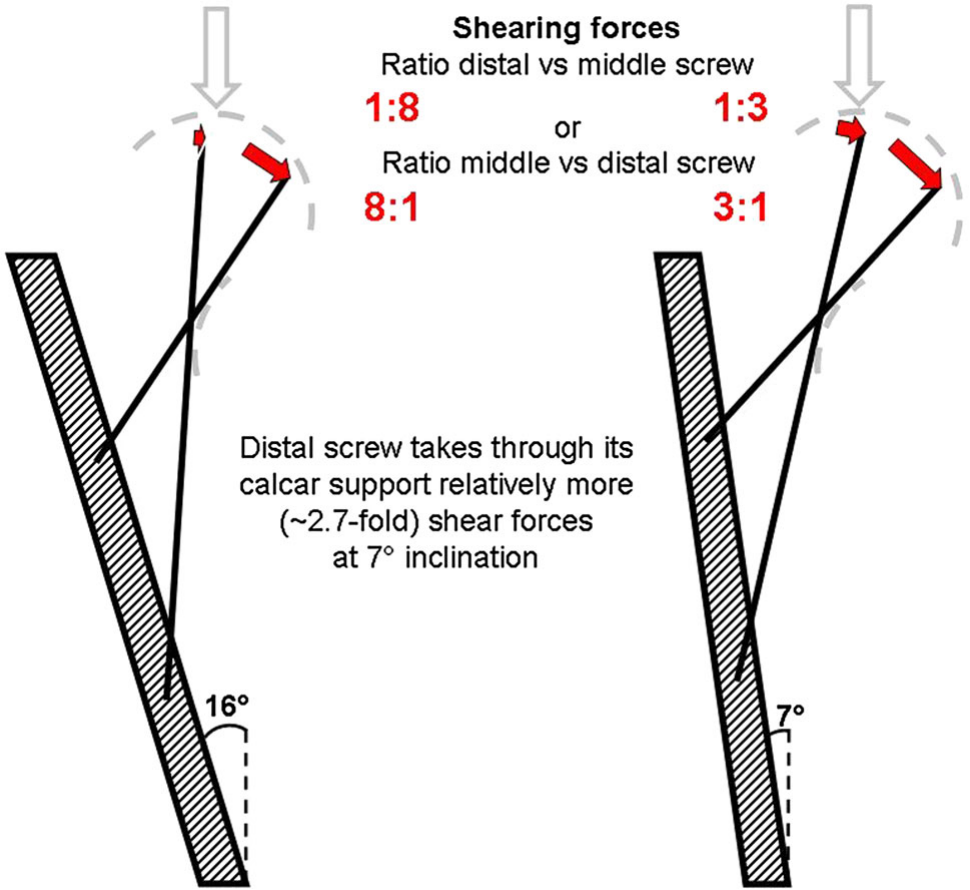
Shearing forces

Actually, such a biomechanical study comparing the novel BDSF method to the traditional inverted triangle parallel screw fixation (CFIX) with the use of human cadaveric femora has been published [1]. It demonstrated about 44% higher axial fixation strength for BDSF in 7° varus inclination compared to conventional parallel screw fixation (initial axial stiffness instrumented, BDSF 0.93 ± 0.10 kN/mm vs. CFIX 0.53 ± 0.06 kN/mm); 15% higher secondary axial stiffness; and 20% higher failure load, and with similar strength in 16° varus inclination. Furthermore, conventional CFIX stability differed significantly between the two inclinations: higher axial stiffness was observed at 16° varus inclination (0.85 kN/mm) vs. 38% lower stiffness in 7° (0.53 kN/mm). In contrast, BDSF stability remained similar at both inclinations. Interestingly, axial BDSF stiffness at the more unstable situation with 7° varus inclination was even higher than that at 16° inclination of the femur. The similar BDSF stability at both inclinations resulted mainly from the presence of a second calcar screw—the distal BDSF screw and its specific position and inclination (Figs. 1, 2, 3).

When loads are oriented more vertically (Fig. 2), closer to the diaphyseal axis, the femoral neck and the screws, which are parallel to the neck axis, became more *horizontal* to the vertical axial load and construct stability is expected to decrease (as observed following conventional CFIX) due to the increasing transverse component of the load acting on the beam construction, which leads to increase of the shearing forces. Mechanically, the *middle* BDSF calcar screw and the only CFIX calcar screw—the distal one, are fairly equivalent and demonstrate similar entry points, calcar support, lengths, and inclinations (parallel to the neck axis). However, in contrast to conventional CFIX, BDSF provides two calcar-buttressed screws that are oriented at different inclinations. If the load is more vertically oriented (Fig. 2), the mentioned *middle* BDSF screw becomes more *horizontal* and decreases its bearing capacity, and the obtuse *distal* BDSF screw comes in optimal orientation for axial weight bearing. Its bearing capacity is added to the middle BDSF screw and helps maintain constant stability across a wide range of inclinations during gait activities, contrary to CFIX.

With double support at the inferior and posterior femoral neck cortices, the distal BDSF screw could be especially effective when axially loaded along the diaphyseal axis and when AP bending and torsion are applied. This is an essential advantage of the BDSF method because during diverse patient activities the resultant dynamic forces and moments change their directions, loading the femoral neck in axial compression (e.g., standing on one leg, standing with the feet apart), AP bending and torsion (e.g., rising up from a chair, climbing, running), where the three parallel CFIX screws, all placed at an angle of 120°–130° to the diaphyseal axis, can be far less functional.

Moreover, the BDSF failure load in the much more unstable situation with 7° varus inclination was similar to that at 16° inclination. This is probably due to the two calcar-buttressed BDSF screws and the specific role of the distal one.

The console-shaped proximal femur demands the screw fixation have to act as a *console beam* with two fulcrum points of support in the distal fragment. The conventional parallel screw fixation has been gold standard for many years. However, determined by the femoral anatomy, when the screws are parallel to one another, the entry points of the screws are located in the fragile cortex of the greater trochanter region, with absence of appropriate second (lateral fulcrum) support for the screws, with the stability relying on the friction between the slipping bone fragments and the lag-screw compression only. Therefore, with the fixation using three parallel screws, due to lack of *two* solid supporting points these implants act as a lever first class (with a medial support only) or as a beam on elastic foundation (without any cortical support), and hardly as a console beam [3]. Therefore, even with a medial cortical support achieved, this type of fixation is associated with up to a 46% rate of complications [4, 5], and patients are usually not allowed full weight bearing immediately [6].

The novel method of Biplane double-supported screw fixation (BDSF) offers better stability by using three medially diverging cannulated screws with two of them buttressed on the calcar. Biomechanically, the most effective component is the distal screw placed at steeper angle and supported on a large area along the distal and posterior cortex of the femoral neck following its spiral anterior curve. Thereby, BDSF achieves the strongest possible distal-posterior cortical support for the fixation construct, which allows for immediate full weight-bearing. In general, compared with conventional parallel screw fixation (CFIX), the fixation strength of BDSF is considerably higher because of the following factors. (1) Two calcar-buttressed screws are used in BDSF, as opposed to only one screw in CFIX. (2) The two calcar screws are in contact with the distal neck cortex in two different regions, located 1–2 cm apart from each other (depending on the CCD angle), and distribute the applied axial load over a larger surface area. Consequently, in contrast to CFIX, the applied load is spread over approximately 50% of the femoral neck cortex length without concentrating stress in a single spot, thereby resulting in increased bearing capacity. (3) The steeper screw orientation angle to the diaphyseal axis contributes to increased varus resistance, reduced beam sagging, and allows for easier sliding when osteoporotic fracture impaction and shortening occurs during weight bearing, thus avoiding cut-out and maintaining stronger fixation strength. (4) Expected reduced risk of subtrochanteric fracture. Due to the specific position of the distal BDSF screw placed at an obtuse angle, this screw receives strong medial support on a large area along the distal and posterior cortex of the femoral neck following its spiral anterior curve from the zone of the basicervical line laterally, up to the convex distal-posterior cortex in the zone of the midcervical line medially. Compared to conventional CFIX, with BDSF the distance between the lateral supporting point (the screw entry in the diaphyseal cortex) and the most medial supporting point (on the mid-cervical line) of the distal BDSF screw is increased because of its steeper angle to the diaphyseal axis. As a result, the load acting on the lateral and medial cortical-supporting points is reduced by 42 and 16%, respectively [3]. Furthermore, the distance between the distal and medial screw entry points is increased to 20–40 mm, allowing for the tensile forces to spread over a larger area on the lateral cortex. (5) The medial support of the distal BDSF screw located on a large area along the distal and posterior cortex of the femoral neck extends laterally up to the zone of the basicervical line of the inferior neck cortex. Therefore, BDSF can be used for the fixation of more unstable fractures with posterior comminution and/or more vertical fracture lines, whereas CFIX would be inappropriate in these situations. (6) Biologically, BDSF screws are positioned in the ventral and dorsal oblique planes, away from the weight-bearing upper pole of the femoral head, and can thereby avoid the danger of damaging the intraosseous vascularisation.

## Follow-up period. Subtrochanteric fractures

Question 2. The reader recommends “Though you did not observe any iatrogenic subtrochanteric fracture after surgery, the average follow-up period was just 29.6 months. It is strongly recommended that additional long-term follow-up studies are needed to further justify its widely use”.

An iatrogenic subtrochanteric fracture after surgery means fracture caused by, or subsequent soon after the surgical intervention, and if iatrogenic, such a fracture should occur immediately after surgery or soon after that, or within the first two months, as a complication of mechanical type. Any follow-up period larger than two months is principally not relevant for registration of mechanical complications [7–11].

Of specific clinical relevance, although the placement of the most distal BDSF screw is below the level of the lesser trochanter, we did not observed any iatrogenic subtrochanteric fracture after surgery in the clinical practice. Some studies recommend that parallel screws should be applied without entering the lateral cortex below the lesser trochanter to prevent of subtrochanteric fracture complication [12, 13]. Admittedly, the small distance of less than 7 mm between the three parallel, cannulated screws placed at 130° may be a significant stress-riser in this area, especially when the distance is further decreased with a steeper angle of placement of the cannulated screws when trying to enter screws below the lesser trochanter level. However, the wider distance between the screw holes in the BDSF method (20–40 mm) might not weaken the subtrochanteric region as the tensile forces acting on the lateral cortex are spread over a larger area. Furthermore, the steeper screw angle leading to larger distance between the fulcrums would further decrease the tension load (i.e., beam theory) [3], as it has been described above. Also, the screw holes placed wide apart from each other could hardly be stress-risers, because they are round, made by drilling and without angular defects.

However, a subtrochanteric fracture is a rare complication even for conventional fixation methods. Furthermore, such a fracture can occur in any side falling with a strong blow on the floor or by a torsion mechanism, and its rate seems the same, both for the femora after BDSF fixation, after conventional parallel screw fixation, and for the intact femora, according to our knowledge.

Although our study is focused on improving the fixation strength which is demonstrated by achieving bone union within 3 months or mechanical and/or biological deficiencies, called with the collective term nonunion, occurred within 6 months, including failure of fixation and pseudoarthrosis [7–11], our mean follow-up period of 29.6 months may be insufficient for registration of all cases of avascular necrosis (AVN) and it has been pointed in our Limitations section.

However, additional long-term follow-up studies investigating BDSF, performed with proper cortical-screw support and screw orientations according to the original operative technique, are expected with great interest. The BDSF method is surprisingly effective and much better than the conventional parallel screw fixation when performed by a trained for this method surgeon and probably could be not so effective in the hands of a surgeon unfamiliar with the method, either experienced or young, as it is true for most surgical methods. It is strange how all arthroplasty procedures, all fractures involving large joints, such as Tibial plateau Schatzker IV–VI fractures, or Tibial pilon fractures are carried by the most experienced in the department senior-surgeons only, but the majority of the femoral neck osteosyntheses are left for young surgeons. We think, such an important large-joint-preserving procedure should be performed by experts only.

## Indications for application of BDSF

Question 3. The reader states: “Fig. 5 shows an eldly patient (79 years old) with distal femoral neck fracture. We don’t choose closed reduction and internal fixation for this kind of patients. Please elaborate your indication of closed reduction and internal fixation for femoral neck fracture”.

Probably, here the reader means femoral neck fracture, not “distal” femoral neck fracture.

Regarding the choice of internal fixation for elderly patients, strong evidence supports the use of arthroplasty for most elderly patients with displaced femoral neck fractures, according to the AAOS recommendations; references [6, 12], and many others. As we have described and discussed in our current study, “Indications for application of BDSF in our practice are fractures of the femoral neck from I to IV stage by Garden, which are generally considered to meet the indications for internal fixation based on accepted clinical algorithms [6, 12]. Accordingly, BDSF can be applied for patients younger than 65 years, for high-demand patients aged more than 65 years without preexisting pathology in the hip joint, for non-ambulatory low functioning patients unfit for arthroplasty, as well as for all patients with non-displaced femoral neck fractures. Fractures of Pauwels type III are contraindicated if they pass laterally of the midcervical line”.

As it is well known, the most of the “non-ambulatory low functioning patients unfit for arthroplasty” are elderly patients, and they could be aged not only 79, as is the cited patient in our Fig. 5, but such patients unfit for arthroplasty could be much older. For example, we have a 102 years old patient with Garden IV fracture (anaesthesiology risk: ASA IV-E), who successfully underwent BDSF-fixation in 2016. Another example could be our 78-year-old patient with Garden IV fracture and with untreated diabetic foot, with gangrene semi-self amputation of the great toe, representing a high septic risk for primary arthroplasty. The patient was lucid, an ex-officer, and due to the great hip pain we preferred internal fixation applying the stronger than conventional screw fixation and only applicable for osteoporotic bone—the BDSF method. After proper disinfection and isolation of the gangrene foot by iodine dressing, we performed BDSF-fixation which, according to our knowledge, is a better approach than firstly to amputate the gangrene foot and to wait its sanation (hip fracture pain for the patient) and to perform arthroplasty as a second step; and also our approach we think is much better than a Girdelstone procedure. In our geriatric orthopaedic institution we have many polymorbid patients and some of them close to the end of their lives, who do not meet any anaesthesiological criteria for the large surgical procedure of primary arthroplasty, and who have been treated by BDSF. Despite the osteoporosis present in some of the patients, 96% of them healed, and 84% healed uneventfully, counting the 12% of AVNs.

Thank you for your consideration.

We thank to all our readers and we encourage and will answer to any questions regarding BDSF application.

The authors.
